# Electroacupuncture Regulates Apoptosis/Proliferation of Intramuscular Interstitial Cells of Cajal and Restores Colonic Motility in Diabetic Constipation Rats

**DOI:** 10.1155/2013/584179

**Published:** 2013-11-18

**Authors:** Juanjuan Xu, Yan Chen, Shi Liu, Xiaohua Hou

**Affiliations:** Division of Gastroenterology, Union Hospital, Tongji Medical College, Huazhong University of Science and Technology, 1277 Jiefang Road, Wuhan, Hubei 430022, China

## Abstract

Injury of interstitial cells of Cajal (ICC) is associated with gut dysmotility in diabetic rats. We have shown an acceleration of the colonic contractility by electroacupuncture stimulation (EAS). However, little is known about potential roles of EAS on colonic transit and ICC. In this study, we evaluate the effect of EAS on colonic transit and investigate whether apoptosis/proliferation of ICC was involved in regulative effect of EAS on colonic transit. Rats were randomly assigned to normal, diabetic, diabetic-plus-sham stimulation, diabetic-plus-low-frequency stimulation, and diabetic-plus-high-frequency stimulation groups. Bead expulsion test was used for measuring the distal colonic transit. The Kit (ICC marker) was detected by western blot. Apoptotic ICC was detected by terminal dUTP nucleotide end labeling. Proliferating ICC was identified by Kit/Ki67 double immunofluorescent staining on whole mount preparations. Ultrastructure changes of ICC were studied using electron microscopy. Results showed that high-frequency stimulation significantly promoted colonic transit. Low- and high-frequency stimulation markedly rescued intramuscular ICC from apoptosis. Abundant proliferating intramuscular ICC was found in low- and high-frequency stimulation groups. Our results indicate that high-frequency EAS has stimulatory effect on the distal colonic transit, which may be mediated by downregulation of the apoptosis and upregulation of the proliferation of intramuscular ICC.

## 1. Introduction

Diabetes-related constipation is one of the most common gastrointestinal (GI) complications of diabetes mellitus (DM). A study found that as many as 60% of patients with DM reported symptoms of constipation or delayed colonic transit [1]. Treatment of diabetes-related constipation is mainly empirical and directs towards symptomatic relief, including good hydration, regular physical activity, and increased fiber intake. However, the therapeutic effect is often not satisfactory.

Numerous studies have been performed to investigate the effects of acupuncture or electroacupuncture on GI motility and functional gastrointestinal diseases [2]. Electroacupuncture is a modification of acupuncture which stimulates acupoints with electrical current instead of manual manipulation [2, 3]. It was found that the effects of electroacupuncture stimulation (EAS) on GI motility were fairly consistent and the major acupoint used in these studies was *Zusanli* (ST-36). Gastric motility has been mostly studied, whereas only a little information is available on the effect of EAS on colonic motility. EAS at ST-36 has been documented to accelerate colonic motility and transit in conscious rats [4]. Our previous study reported that EAS at ST-36 can promote distal colonic contractility via a cholinergic pathway in conscious rats [3]. Rangwala et al. has showed that EAS is a safe and effective method for treatment of constipation without any adverse effect [5]. Recently, our study showed that EAS at acupoint ST-36 can increase the density of interstitial cells of Cajal (ICC) and upregulate the expression of colonic ICC [6]. However, the potential roles of EAS on colonic motility and ICC in diabetic constipation rats have scarcely been investigated. 

The important role of ICC in gastrointestinal physiology has been elucidated over the past 10–20 years. ICC is necessary for normal intestinal motility [7]. Intramuscular interstitial cells of Cajal (ICC-IM) were primarily affected in DM, which was associated with delayed colonic transit [8, 9]. Further studies [10, 11] have found that the network of ICC is not static, and it goes through periodical changes even under normal physiological conditions. Apoptosis and transdifferentiation can lead to ICC deletion; ICC can be restored by proliferation, replenishment from stem cells, and increasing survival; whether apoptosis/proliferation of ICC is involved in the effect of EAS on colonic transit is unclear. 

This paper hopes to provide more data for EAS on GI tract in diabetic rats and the underlying mechanism. The aims of our study were to evaluate the effect of EAS at ST-36 on colonic transit in diabetic constipation rats and to investigate whether apoptosis/proliferation of ICC was involved in regulative effect of EAS on colonic transit.

## 2. Materials and Methods

### 2.1. Animals

Fifty male Sprague-Dawley rats, weighing 250–300 g were used in the present study. The rats were purchased from Tongji Medical College and housed under normal laboratory conditions (18–22°C, 12/12 h light-dark cycle) with free access to food and water *ad libitum*. This study was started after the rats were adapted to the environment, usually after one week. Rats in our study received humane care and were treated strictly in accordance with the recommendations in the Guide for the Care and Use of Laboratory Animals of the National Institutes of Health, and all experimental works were approved by the ethical guidelines from Animal Care and Ethics Committee of Tongji medical college, Huazhong University of Science and Technology (number 00008371).

### 2.2. Induction of Diabetes

Diabetes was induced with a single intraperitoneal injection of streptozotocin (Alexis Biochemicals, San Diego, CA, USA) 60 mg kg^−1^ freshly dissolved in 20 mmol L^−1^ citrate buffer solution (Sigma, St. Louis, MO, USA). Age matched controls were injected with an equal volume of citrate buffer. Blood glucose and body weight were measured prior to injection as well as 1 week, 4 weeks, and 8 weeks after injection. Diabetes was verified by a random blood glucose reading higher than 16.7 mmol L^−1^ after injecting streptozotocin (STZ) for one week, measured using blood glucose meter on blood taken from the tail vein.

### 2.3. Experimental Protocols

 Animals were randomized into five groups (ten rats/group), including the control group, the DM group, the DM-plus-sham stimulation group (SEA, only acupuncture but no electrical current, 30 min), the DM-plus-low-frequency stimulation group (LEA, 10 Hz, 1–3 mA, 30 min), and the DM-plus-high-frequency stimulation group (HEA, 100 Hz, 1–3 mA, 30 min). EAS was performed at ST-36 daily for 8 weeks [3]. The distal colonic transit was recorded prior to injection as well as 1 week, 4 weeks, and 8 weeks after injection. Later, rats were subsequently sacrificed, and specimens of the distal colon were obtained from each constipated rat. Each specimen collected from the rats was divided into three pieces of equal size, which were then either immediately removed from the mucosa and submucosa, and stored at −80°C for western blot analysis or placed into 4% paraformaldehyde for immunofluorescence, or 2.5% glutaraldehyde for electron microscopy.

### 2.4. Electroacupuncture

ST-36 was located at 5 mm lateral and inferior to the anterior tubercle of the tibia in rats [3]. Needles of 0.3 mm in diameter (Suzhou Medical Appliance Factory, Jiangsu, China) were inserted into the acupoints, which were stimulated by electroacupuncture with parameters of 10 Hz and 100 Hz and 1–3 mA using an electrical stimulator (G6805-2A; Shanghai Huayi Medical Instrument Factory, Shanghai, China). These parameters were selected based on the preliminary experiments which suggested a stimulatory effect of motility [4, 12]. In order to exclude the influence of restraint condition, animals were allowed to move freely in their own cage during the EA procedure and motility recording.

### 2.5. Measurement and Analysis of Colonic Motility

A modification of techniques used previously [13, 14] was employed in this study to assess the distal colonic motility. Animals were fasted for 12 hours before the measurements. Briefly, a single 3 mm colored plastic bead was inserted into the distal colon (3 cm past the anus) with a plastic rod, while each animal was under brief isoflurane anesthesia. Bead insertion was accomplished using a glass rod with a fire-polished end to avoid tissue damage. After bead insertion, the conscious rats were placed individually into a cage with a white paper towel covering its floor to allow for easier detection of the bead. Distal colonic transit was determined as the time between bead placement and expulsion of the bead (bead latency). The latency to the expulsion of the bead was recorded. This was repeated three times. Rats with a bead expulsion time exceeding $\bar{\text{X}}$ + 2SEM were considered to be constipated. The number of rats in control group is ten, and the number of diabetic constipation rats in each other group is listed as follows: (i) six rats in DM group, (ii) seven rats in SEA group, (iii) seven rats in LEA group, and (iv) seven rats in HEA group.

### 2.6. Western Blot

 Protein concentrations were analyzed by the BCA reagent (Pierce, Rockford, IL, USA). One hundred micrograms of protein was separated by 10% sodium dodecylsulfate-polyacrylamide gel electrophoresis and transferred to PVDF membrane (Millipore, Bedford, MA, USA). Immunoblotting using the primary antibodies against Kit (1 : 200, sc-168-G, Santa Cruz Biotechnology, Inc.) was done; rabbit anti-rat actin (1 : 400, Santa Cruz Biotechnology, Inc.) was used as internal control, followed by detection with enhanced chemiluminescence (Amersham Pharmacia Biotech, Piscataway, NJ, USA) and quantified by densitometry.

### 2.7. Immunofluorescence

The distal colon was removed gently and enteric contents were washed away with PBS (37°C) after the abdomen was opened. For whole-mount preparations, the colon was inflated with fixation fluid, the mucosa and submucosa were then removed, and the circular smooth muscle layer containing the ICC-IM was exposed with the aid of a dissection microscope. The whole-mount preparations were incubated in normal bovine serum (5% in PBS containing 0.3% Triton X-100) for 1 h at room temperature and then incubated firstly with primary antibody Kit (goat anti-rat, 1 : 150; Santa Cruz Biotechnology, Inc., Santa Cruz, CA, USA) overnight at 4°C, and then with secondary antibodies (anti-goat, 1 : 100; Alex Fluor). The stained results were detected by confocal laser scanning microscope (Nikon, Japan) with an excitation wavelength appropriate for FITC (488 nm, proliferation) and Cy3 (552 nm, apoptosis). The Z stacking of confocal images at 3 *μ*m to 5 *μ*m intervals contained all the levels of positively stained cells and processes.

### 2.8. Detection of Apoptosis ICC

To identify apoptosis of ICC, terminal deoxynucleotidyl transferase-mediated dUTP nick end labeling (TUNEL) method was done using an apoptosis detection kit (Roche, Germany) [15]. Whole-mount preparations were incubated with primary antibody Kit (goat anti-rat, 1 : 150; Santa Cruz Biotechnology, Inc., Santa Cruz, CA, USA) overnight at 4°C and secondary antibody (anti-goat, 1 : 100; Cy3; Alex Fluor) at 37°C for 30 min, washed twice with PBS for 30 min, and then were incubated with TUNEL reaction buffer containing 45 *μ*L label solution and 5 *μ*L enzyme solution (labeling solution : enzyme solution = 9 : 1) at 37°C for 1 hour in a humidified atmosphere in the dark. Washed with PBS for three times (each 15 min) to remove unincorporated fluorescein-dUTP. The specimens were observed with a fluorescence microscope with an excitation wavelength in the range of 450–500 nm. 

### 2.9. Detection of ICC Proliferation

To detect the proliferation of ICC, anti-Ki67 antibody was used to detect the presence of proliferative ICC. Ki67 is a nuclear protein that is expressed in proliferating cells and may be required for maintaining cell proliferation. The whole-mount preparations were stained for Kit as described above and then labeled for Ki67 as follows: whole-mount preparations were incubated with a rabbit monoclonal Ki67 antibody (rabbit anti-rat, 1 : 200; Abcam, Cambridge, UK) overnight at 4°C and then with a cy3-conjugated secondary antibody (anti-rabbit, 1 : 100; Alex Fluor). The specimens were observed with a fluorescence microscope with an excitation wavelength appropriate for Cy3 (552 nm). 

### 2.10. Electron Microscopy

The distal colon was perfused with PBS followed by fixative solution (2.5% glutaraldehyde). Then, they were immersed for 2 h at 4°C in the 2.5% glutaraldehyde. Tissue sample fragments of about 1 mm × 5 mm were cut from the body of distal colon and were fixed with 1% OsO_4_ for 30–120 min. They were washed with 0.1 M phosphate buffer and dehydrated in a series of graded ethanol. After immersion in propylene oxide, the samples were immersed in mixtures (1 : 1) of propylene oxide and Epon resin for 2 h, and embedded in Epon according to the instruction sheet. The regions to be studied were identified and sectioned using an ultramicrotome into ultrathin (70 nm) serial sections. We visualized the sections using a transmission electron microscope operating (Tecnai G212, FEI, The Netherlands).

### 2.11. Measurement and Statistical Analysis

 After immunofluorescent staining was assessed, photomicrographs of both Kit/TUNEL and Kit/Ki67 double-labeled cells were taken in ten random fields (200× magnification, 0.2607 mm^2^) per whole-mount preparation with a digital camera (SPOT, Diagnostic Instruments) in a BX51 fluorescence microscope (OLYMPUS, Japan). The numbers of either Kit/TUNEL or Kit/Ki67 positive cells were independently counted by two observers with Image-Pro plus 5.0 (Media Cybernetics). All data were presented as mean values ± SEM. One way analysis of variance (ANOVA) was used for multiple group comparisons, and *P* < 0.05 was taken as a statistically significant difference. 

## 3. Results

### 3.1. Blood Glucose and Body Weight

**Figure 1:**
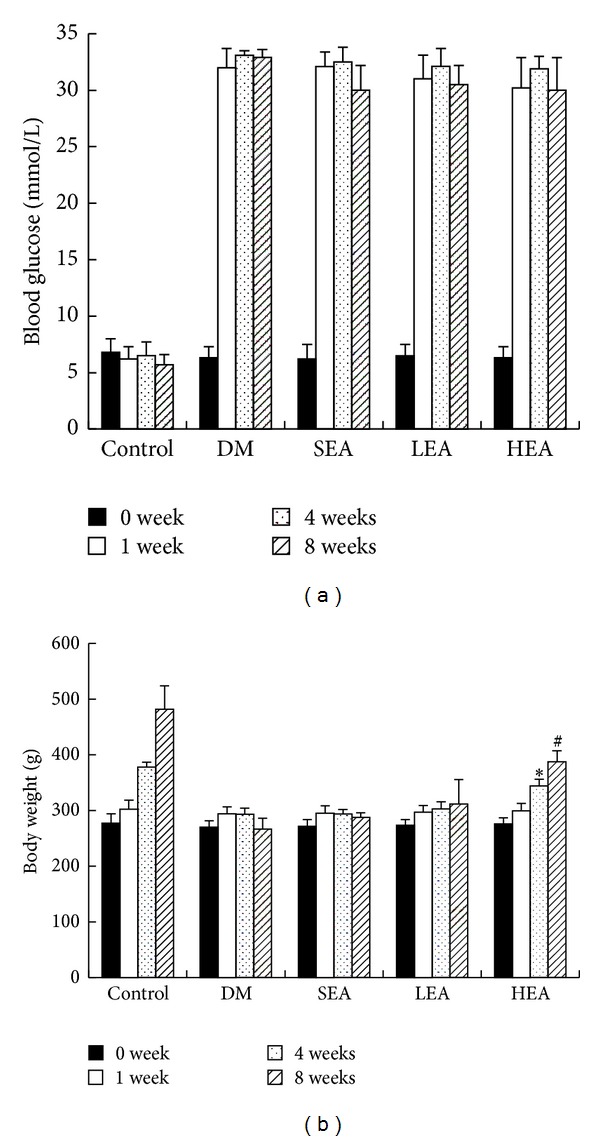
Blood glucose (a) and body weight (b) in the 5 groups. (a) All the diabetic rats displayed markedly increased blood glucose. In the SEA, LEA, and HEA groups, blood glucose did not show any changes compared with the DM group (*P* > 0.05). (b) No differences in the baseline body weight among each group. The HEA group gained weight compared with the DM group at the end of 4 weeks (*P* < 0.01) and 8 weeks (*P* < 0.01). (*Significantly different to the DM group for 4 weeks, ^#^significantly different to the DM group for 8 weeks.)

As shown in Figure 1(a), there were no differences in the baseline blood glucose among each group. At the end of 4 and 8 weeks, blood glucose level in the untreated diabetic rats was markedly higher than that of the controls (*P* < 0.01 and *P* < 0.01, resp.). In the SEA, LEA, and HEA groups, blood glucose levels did not show any significant changes compared with the DM group for 4 weeks (*P* > 0.05, > 0.05, and > 0.05, resp.) and 8 weeks (*P* > 0.05, > 0.05, and > 0.05, resp.).

 Otherwise, no differences in the baseline body weight among each group (Figure 1(b)). At the end of 4 and 8 weeks, the body weight of the rats in the DM group did not increase, whereas the HEA group gained weight (*P* < 0.01 and *P* < 0.01, resp.). In the SEA and LEA groups, body weight was not significantly changed compared with the DM group for 4 weeks (*P* > 0.05 and > 0.05, resp.) and 8 weeks (*P* > 0.05 and > 0.05, resp.).

### 3.2. Effects of EAS on Distal Colonic Transit

**Figure 2:**
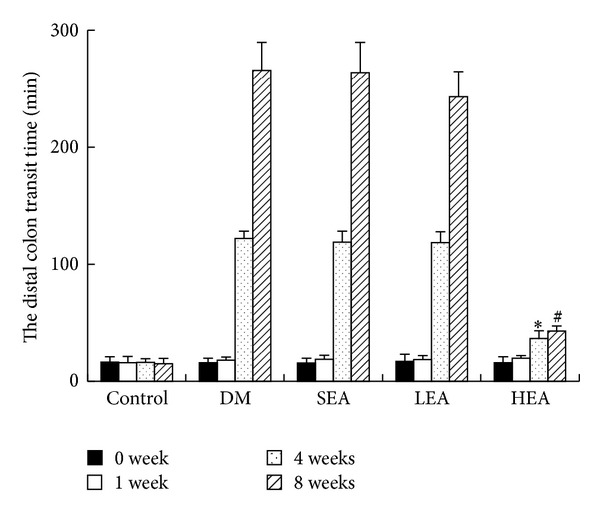
Effects of EAS at ST-36 on distal colonic transit. In contrast to the control, the DM group displayed delayed colonic transit, while EAS with high-frequency at ST-36 significantly increased colonic transit. The colonic transit time was not changed obviously in the SEA and LEA group. Data are given as means ± SEM; an asterisk indicates statistical significance at a level of *P* < 0.05. (*Significantly different to the DM group for 4 weeks, ^#^significantly different to the DM group for 8 weeks.)

As shown in Figure 2, no differences were noted in the baseline colonic transit time among the five groups. At the end of 4 and 8 weeks, the distal colonic transit in the DM group was delayed significantly (122.0 ± 6.4 min versus 16.1 ± 3.2 min, *P* < 0.01; 265.7 ± 23.9 min versus 15.1 ± 4.5 min, *P* < 0.01). In the SEA and LEA group, the distal colonic motility was not significantly altered compared with the DM group for 4 weeks (119.0 ± 9.3 min versus 122.0 ± 6.4 min, *P* > 0.05; 118.6 ± 9.3 min versus 122.0 ± 6.4 min, *P* > 0.05) and 8 weeks (263.6 ± 25.9 min versus 265.7 ± 23.9 min, *P* > 0.05; 243.2 ± 21.3 min versus 265.7 ± 23.9 min, *P* > 0.05), respectively. However, the HEA at ST-36 significantly promoted the distal colonic motility for 4 and 8 weeks, respectively (36.7 ± 6.6 min versus 122.0 ± 6.4 min, *P* < 0.01; 43.1 ± 4.2 min versus 265.7 ± 23.9 min, *P* < 0.01).

### 3.3. Western Blot Analysis of Kit in Colon Tissue from Which the Mucosa and Submucosa Were Removed

**Figure 3:**
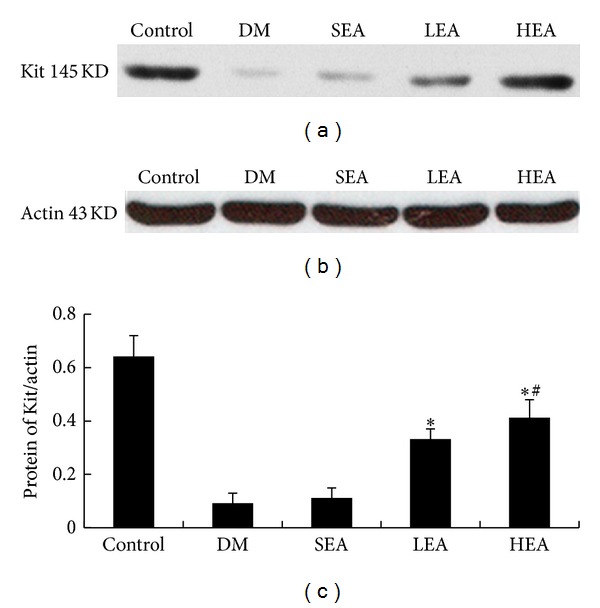
Western blot analysis of Kit in colon tissue which the submucosa removed. Compared with the control group, the expression of Kit was decreased in the DM group; there were no significant difference between the DM and SEA group (*P* > 0.05). However, they were increased markedly in the LEA and HEA group. (*Significantly different to the DM group, ^#^significantly different to the LEA group.)

In contrast to the control group, the expression of Kit in the DM group was decreased markedly (*P* < 0.01). Similarly, no significance was detected between the SEA and DM group (*P* > 0.05). In the LEA group, the expression of Kit showed a significant increase (*P* < 0.01), while, in the HEA group, an enhancement was found nearly four times higher than the DM group. The increased level of Kit expression in the HEA group was somewhat higher than the LEA group, and the difference was statistically significant (*P* < 0.01) (Figure 3).

### 3.4. Changes in ICC-IM Density

ICC-IM was found within the smooth muscle layers of the colon; cells were revealed by immunohistochemistry of Kit in confocal whole-mount preparations that clearly showed ICC-IM forming a cellular network in control group. In the DM group, the density of Kit positive ICC-IM was significantly reduced by ~78% compared with the control (1.3 ± 0.5 versus 5.9 ± 0.7 × 10^2^ mm^−2^, *P* < 0.01). However, Kit^+^ cell numbers had partly restored in the LEA and HEA group, and almost intact cellular networks could be observed. Kit positive cell density in the LEA group was still significantly lower than that in the HEA group (3.3 ± 0.5 versus 3.9 ± 0.2 × 10^2^ mm^−2^, *P* < 0.05). There was no difference between the SEA and DM group (1.7 ± 0.4 versus 1.3 ± 0.5 × 10^2^ mm^−2^, *P* > 0.05).

### 3.5. Apoptosis of ICC-IM

**Figure 4:**
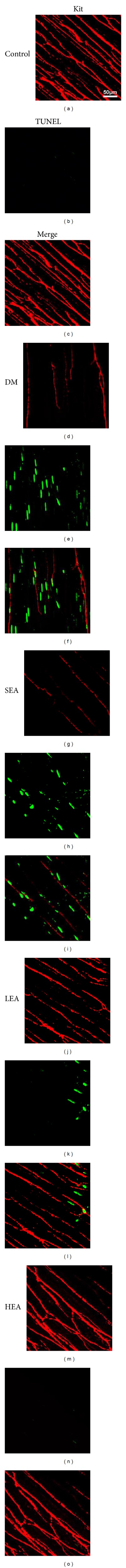
Confocal images of ICC-IM in each group showing the apoptosis of ICC-IM. (a)–(c) In the control group, nearly no cell was labeled by TUNEL method and displayed a perfect network. (d)–(f) In the DM group, a large number of Kit^+^/TUNEL^+^ cells (*arrowheads*) were observed, and the Kit^+^ cellular networks were severely damaged. (g)–(i) In the SEA group, a number of apoptotic Kit^+^ ICC-IM (*arrowheads*) were observed and the network of ICC-IM was not restored. (j)–(l) In the LEA group, the network of ICC-IM was partially restored and rare double labeled cells (*arrowheads*) were distributed within ICC-IM. (m)–(o) In the HEA group, the apoptotic ICC-IM nearly vanished and was accompanied by a more intact network. Scale bar = 50 *μ*m and refers to all panels.

In order to determine whether apoptosis was involved in the process of ICC-IM loss in diabetes, TUNEL method was used to detect the apoptosis of ICC (Figure 4). In the control group, nearly no cell labeled by TUNEL method was found in the distal colon wall on whole-mount preparations, and the Kit^+^ ICC-IM networks were perfect. In the DM group, the Kit^+^ cellular networks were severely damaged, and a large number of Kit^+^/TUNEL^+^ cells were observed which were located within the ICC-IM (Table 1). Similar change was seen in the SEA group, and a number of apoptotic Kit^+^ ICC were observed (*P* > 0.05). However, in the LEA and HEA group, the network of ICC was restored; a more intact network was seen in the HEA group, and rare apoptotic ICC could be seen. 

### 3.6. Proliferation of ICC-IM

**Figure 5:**
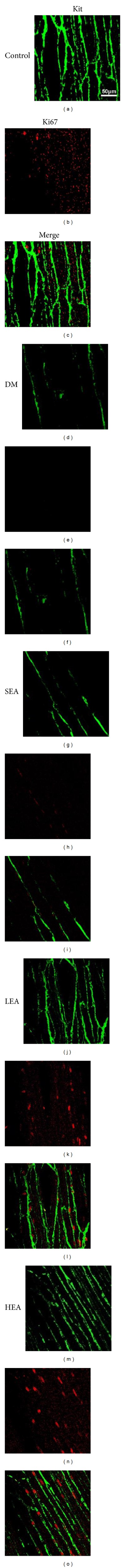
Confocal images of ICC-IM in each group showing the proliferation of ICC-IM. (a)–(c) In the control group, a small number of proliferative ICC-IM (*arrowheads*) were observed and displayed a perfect network. (d)–(f) In the DM group, Kit^+^/Ki67^+^ were almost absent, and the network was severely damaged. (g)–(i) In the SEA group, there was also no proliferative ICC-IM and the network of ICC-IM was not restored. (j)–(l) In the LEA group, the network of ICC-IM was partially restored and a small number of double labeled cells (*arrowheads*) were distributed within ICC-IM. Such cells were in high density in the HEA group (m)–(o), accompanied by a more intact network. Scale bar = 50 *μ*m and refers to all panels.

In view of significant increase of the ICC-IM cell number in the LEA and HEA group, we determine whether proliferation was involved in the dramatic increase in cell number. Therefore, double immunofluorescent staining with anti-Kit/Ki67 was used to reveal the presence of ICC proliferation. Ki67 is a nuclear protein which is expressed in proliferating cells and maybe essential for maintaining cell proliferation [16]. A small number of proliferative ICC appeared in the control group and displayed a perfect network. Kit/Ki67 ICC-IM like cell labeling was neither seen in the DM group nor in the SEA group. However, the network of ICC-IM was partially restored and more double labeled cells were distributed within ICC-IM in the LEA group (11.9 ± 1.0 mm^−2^). Such cells were presented in high density in the HEA group (13.1 ± 0.9 mm^−2^, Table 1) and accompanied by a more intact network of ICC (Figure 5). 

### 3.7. Electron Microscopy: Ultrastructure of ICC-IM

**Figure 6:**
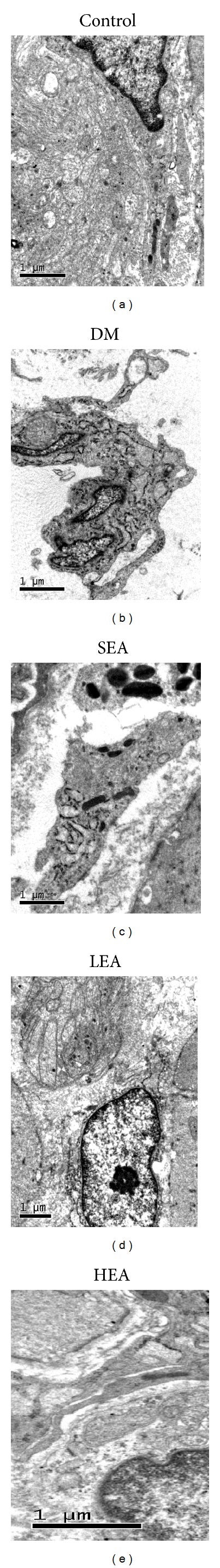
Ultrastructure of ICC. Comparing with the control group, ICC was seriously injured in the DM and SEA group, while they showed nearly normal structure and minor injury in the LEA and HEA group. ICC: interstitial cells of Cajal; SMC: smooth muscle cell; NF: nerve fibre.

In the control group, ICC-IM had a higher electron-density cytoplasm compared with smooth muscle cells and was rich in cell organelles, including mitochondria, endoplasmic reticulum, and basal lamina (Figure 6(a)). They also displayed close connection with enteric nerves and smooth muscle cells. However, ICC-IM in the DM group was markedly affected (Figure 6(b)): swollen mitochondria with disrupted cristae, lamellar bodies, and partial cytoplasmic depletion were frequently present within the cell bodies or processes of ICC-IM. Most of them lost connection with enteric nerves. ICC-IM in the SEA group displayed the same ultrastructural abnormalities as in the DM group (Figure 6(c)). In contrast, most ICC-IM in the LEA (Figure 6(d)) and HEA groups (Figure 6(e)) were restored largely, and occasionally minor injury could be seen.

## 4. Discussion

The present study demonstrated that (i) HEA at ST-36 has stimulatory effect on the distal colonic transit in diabetic constipation rats; (ii) both LEA and HEA can downregulate the apoptosis and upregulate the proliferation of ICC-IM.

Acupuncture has been used to treat a variety of GI problems in China for thousands of years. Electroacupuncture has been more frequently used in the clinical and research setting because of its reproducibility. The advantages of electroacupuncture are that it is a cost-effective and minimally invasive procedure with a very low incidence of side effects. Despite its well-established effects on gastric motility, much less information is available on the effect of EAS on colonic motility. Iwa et al. demonstrated that EAS (10 Hz) at ST-36 accelerated colonic motility and transit in conscious rats [4]. Our previous study has shown an acceleration of the colonic contractility by EAS (10 Hz) at ST-36 in conscious rats [3], and high-frequency EAS (100 Hz) can markedly increase the colonic transit obviously which was published in Chinese data. Other studies also suggested that electroacupuncture, especially high-frequency EA, has usually been used as an alternative therapy for Parkinson disease (PD) and is effective for alleviating motor symptoms in patients and PD models [12]. So, we selected the parameters (10 Hz and 100 Hz) based on the preliminary experiment that showed stimulatory effect of motility. In the current study, we demonstrated that EAS with high-frequency at ST-36 stimulated the distal colonic motility of diabetic constipation rats. While there were no significance changes in low-frequency group, the possible explanation for the different changes of colonic motility respond to low-frequency EAS in different studies may be that rats in the state of disease response differently to EAS, while the possible reason of colonic motility responding differently to low and high-frequency EAS in our study may be that the energy of low-frequency EAS is not enough to cause changes in movement.

In addition to investigating the effect of EAS on distal colonic motility, the experiment was also designed to study the possible mechanisms. Most of the work in this field was focused on outer peripheral vagus nerve system, which suggested that the stimulatory effect may be mediated through the cholinergic pathway [3, 4], and only a few studies reported the effect of EAS on ENS. However, it is widely accepted that the network of ICC plays a very important role in regulation of GI motility. ICC-IM, which form gap junctions with smooth muscle cells and make close connections with nerve varicosities, mediate enteric motor neurotransmission. Wang et al. reported that ICC-IM was primarily affected in diabetes [9]. Forrest et al. found that in the colon, ICC-IM as well as ICC-SMP were affected most severely [8]. Moreover, our recent studies have revealed that EAS can increase the density of ICC and repair the nervous apparatus [6, 17], so we choose to observe the alteration of ICC-IM based on the preliminary experiment. In the present study, we also showed that the density and ultrastructure of ICC-IM were elevated significantly in the EAS group, especially the HEA group, which may be responsible for its efficacy in regulating colonic motility.

Apoptosis and transdifferentiation are the two mechanisms for ICC loss, which is associated with motility disorder [10]. In the STZ model of diabetes, increased apoptosis of neurons in the colon tissue has been demonstrated [18]. Data from human colonic tissue suggest that a decrease in number of ICC in response to injury may occur, at least in part, through apoptosis [19]. We have showed cell death by using TUNEL method and found that a large number of apoptotic Kit positive cells occurred in the DM group, suggesting that a reduction of ICC may be due to apoptosis, at least in part. Wang et al. [20] reported that EAS pretreatment could anti-apoptosis to protect against ischemic damage in the rats of focal cerebral ischemia. In our study, it was interesting to observe that the number of apoptotic ICC-IM in the EAS groups, especially the HEA group, was largely reduced. The results indicated that high-frequency electroacupuncture was more effective in reducing the apoptosis of ICC-IM, and the reduction of apoptosis of ICC-IM might be involved in the mechanism of HEA on colonic motility. 

Expansion of ICC can be achieved by increasing the proliferation of ICC, by development from stem cells, and by an increase in the survival of the remaining ICC [10]. Although the following two mechanisms cannot be discarded, other study suggested that EAS can enhance cell proliferation in young rat brain [21]. Proliferation is involved in the expansion of ICC cell number in the ischemia and reperfusion injury in adult guinea pigs [15], so we study mainly the first mechanism. By using Ki67 incorporation, we demonstrated that there is a recovery of ICC-IM cells number and proliferation in ICC-IM in the EAS groups, especially the HEA group. The results suggested that high-frequency electroacupuncture was more effective in increasing the proliferation of ICC-IM, and increased proliferation of ICC-IM may be involved in the mechanism of HEA on colonic motility.

Hyperglycemia and weight loss were the two main parameters that characterized the diabetic rats [14]. A literature [22] reported that EAS could increase the appetite, sleep, and body weight. In our study, diabetic rats treated with high-frequency stimulation exhibited an increase in body weight, but blood glucose levels after treatment with different modalities of EAS were mostly unchanged, and they were still hyperglycemic. Peplow and Baxter [23] found that EAS of 15 Hz with 10 mA for 30 minutes and 60 minutes had a hypoglycemic effect in fasted type 2 diabetic rats. However, blood glucose was not altered following EAS with parameters of 15 Hz with 1–3 mA in type 2 diabetic rats and 20 Hz in type 1 diabetic rats [23, 24]. Similarly, although a different frequency EAS (10/100 Hz, 1–3 mA) was tried, blood glucose in STZ-induced diabetic rats was not significantly altered in the present study. The possible explanation for the different changes of blood glucose response to EAS in different studies may be that the frequency of EAS and the animal model were different. Our results indicate that HEA can increase the body weight of diabetic rats but has no effect on blood glucose in STZ-induced diabetic rats, a model of type 1 diabetes.

The data of this present research suggest that the effect of EAS at acupoint ST-36 on distal colonic motility is stimulatory, and the stimulatory effect may be mediated by downregulation of the apoptosis and upregulation of the proliferation of ICC-IM. Based on the results of the research, EAS could be applied for treating the colonic motility disorders. In the current study, both low- and high-frequency EAS were capable of improving structural parameters, but functional improvement was only seen after high-frequency EAS, suggesting that the high-frequency EAS may be better to treat the colonic motility disorders. There are studies that have suggested a therapeutic potential of acupuncture for functional constipation [25]. A clinical study has showed that acupuncture can significantly increase the frequency of bowel movements in constipated children [26]. Further studies of enlarged sample and a different frequency EAS are necessary to prove its clinical potential.

## 5. Conclusion

 In conclusion, HEA at ST-36 can promote distal colonic motility, and the stimulatory effects of HEA at ST-36 on distal colonic motility may be, in part, mediated by downregulation of the apoptosis and upregulation of the proliferation of ICC-IM. Further studies are needed to investigate the clinical potential for the treatment of functional gastrointestinal disease. 

## Figures and Tables

**Table 1:** Mean density of Kit^+^/TUNEL^+^ and Kit^+^/Ki67^+^ cells in the ICC-IM in the STZ-induced diabetic model.

	Control	DM	SEA	LEA	HEA
Kit^+^/TUNEL^+^ cells (mm^−2^)	1.0 ± 0.7	19.0 ± 2.4	18.3 ± 2.1	4.0 ± 1.4*	1.1 ± 0.4^∗#^
Kit^+^/Ki67^+^ cells (mm^−2^)	3.8 ± 0.8	0.8 ± 0.4	0.7 ± 0.5	11.9 ± 1.0*	13.1 ± 0.9^∗#^

Data given as mean ± SEM.

*Significantly different from the DM group (e.g., HEA versus DM) (*P* < 0.05).

^#^Significantly different from the LEA group (e.g., HEA versus LEA) (*P* < 0.05).
